# Practical Considerations for Using the NeoSpectra-Scanner Handheld Near-Infrared Reflectance Spectrometer to Predict the Nutritive Value of Undried Ensiled Forage

**DOI:** 10.3390/s23041750

**Published:** 2023-02-04

**Authors:** Xiaoyu Feng, Jerry H. Cherney, Debbie J. R. Cherney, Matthew F. Digman

**Affiliations:** 1 Department of Agricultural and Biosystems Engineering, North Dakota State University, Fargo, ND 58105, USA; 2 Section of Soil and Crop Sciences, School of Integrative Plant Science, Cornell University, Ithaca, NY 14850, USA; 3 Department of Animal Science, Cornell University, Ithaca, NY 14850, USA; 4 Department of Biological Systems Engineering, University of Wisconsin, Madison, WI 53706, USA

**Keywords:** Zea mays, Medicago sativa, portable NIRS, feed composition

## Abstract

Prediction models of different types of forage were developed using a dataset of near-infrared reflectance spectra collected by three handheld NeoSpectra-Scanners and laboratory reference values for neutral detergent fiber (NDF), in vitro digestibility (IVTD), neutral detergent fiber digestibility (NDFD), acid detergent fiber (ADF), acid detergent lignin (ADL), crude protein (CP), Ash, and moisture content (MO) from a total of 555 undried ensiled corn, grass, and alfalfa samples. Data analyses and results of models developed in this study indicated that the scanning method significantly impacted the accuracy of the prediction of forage constituents, and using the NEO instrument with the sliding method improved calibration model performance (*p* < 0.05) for nearly all constituents. In general, poorer-performing models were more impacted by instrument-to-instrument variability. The exception, however, was moisture content (*p* = 0.02), where the validation set with an independent instrument resulted in an RMSEP of 2.39 compared to 1.44 where the same instruments were used for both calibration and validation. Validation model performance for NDF, IVTD, NDFD, ADL, ADF, Ash, CP, and moisture content were 4.18, 3.86, 6.14, 1.10, 2.75, 1.42, 2.71, and 1.67 for alfalfa-grass silage samples and 3.22, 2.21, 4.55, 0.38, 2.07, 0.50, 0.51, and 1.62 for corn silage, respectively. Based on the results of this study, the handheld spectrometer would be useful for predicting moisture content in undried and unground alfalfa-grass (R^2^ = 0.97) and corn (R^2^ = 0.93) forage samples.

## 1. Introduction

Ruminant health and performance depend on adequate and consistent nutrition. Whereas on-farm forages are cost-effective, the nutritive value varies due to weather, soil fertility, and the management of harvest and storage systems [1,2,3]. Consequently, the day-to-day variation of forage nutritive value has been associated with changes in livestock body condition [4]. 

To manage variation in forage nutritive value, producers can supplement the forage with grains and byproducts from the food processing industry. Although these ingredients are typically less variable than forages, their portion of the diet must be balanced with the nutrition provided by the forage, or overfeeding can occur. Overfeeding increases the cost of the diet and can be detrimental to the environment as excess nutrients are excreted in the manure. As such, the diet is monitored by a nutritionist who samples on-farm feeds to send for compositional analyses at an analytical laboratory specializing in agricultural product testing. Those data are used in ration balancing software to ensure adequate and cost-effective nutrition.

Whereas these laboratories are equipped with an array of analytical equipment, near-infrared reflectance spectroscopy has become a cost-effective method to provide multiple chemical species in one analysis. Additionally, spectra gleaned from forage samples have been utilized to predict the outcome of digestibility assays used to estimate animal performance [5]. Though NIRS instrument technology has evolved, the scanning monochromator, specifically the FOSS NIRSystem 6500 (1100–2500 nm, 2 nm step, FOSS, Hillerod, Denmark), remains one of the most common laboratory instruments in the feed and forage industry. 

With the FOSS as the standard, researchers have compared the performance of new instrument designs with favorable results. Bec et al. [6] provide a review of contemporary hardware designs. Fernández et al. [7] demonstrated that PLS models developed on a FOSS instrument could be transferred to a micro-electro-mechanical systems (MEMS) spectrometer (1600–2400 nm, 8 nm step, PHAZIR, Polychromix, Inc., Wilmington, MA, USA) after interpolation to the same step size and piecewise discriminant standardization. Their work presented performance data for predicting feed constituents, including fat, fiber, protein, and starch. A validation set predicted on both instruments demonstrated that the transferred model predicted the constituents with an average increased precision of 0.11% dry matter (DM).

Acosta et al. [8] compared the performance of the FOSS to the PHAZIR and a NIRscan Nano evaluation module (900–1700 nm, 5 nm step, Texas Instruments, Austin, TX, USA). The NIRscan employs a digital micromirror device to focus diffracted light onto a single InGaAs photodetector. To compare the instruments, they developed calibrations to predict crude protein, neutral detergent fiber, acid detergent fiber, and in vitro true digestibility in 210 samples of dried and ground switchgrass and bermudagrass species. Averaging the differences between the FOSS and the PHAZIR and NIRscan across the forage constituents yields an increased precision of 0.4 and 0.9% DM for the PHAZIR and NIRscan, respectively. This work demonstrated that forage constituents could be predicted independently of instrument resolution and range. This supports the known NIRS overtone and combination band physics of constituent diatomic molecules (C-H, O-H, and N-H) in forage chemistry.

Berzaghi et al. [9] compared the FOSS instrument to the NIRscan Nano and a SCiO (740–1070 nm, 1 nm, Consumer Physics, Tel-Aviv, Israel) for a large set of alfalfa (n = 612) and grass samples (n = 516). Forage quality parameters included acid detergent fiber, amylase-treated neutral detergent fiber, 48 h in vitro digestibility, and neutral detergent fiber digestibility. They also evaluated PLS models that used a trimmed dataset to account for instrument range differences. It is common practice in developing forage quality prediction models to restrict the FOSS wavelength range from 400–2500 nm to 1100–2500 nm as the information in the visible range reduces model performance. Averaged across crop species and forage quality parameters, models developed with the full wavelength range reduced precision by 0.05%DM. Comparing the FOSS to the NIRscan resulted in a loss of precision of 0.7%DM, but when the wavelength range between the instruments was matched, that was reduced to 0.3%DM. These numbers are in the same order of magnitude as [8], even though there are differences in the number of samples and the forage quality parameters observed.

Similarly, the SCiO had worse precision compared to the FOSS with an average increase in precision of 1.8%DM, but that was reduced to 0.19%DM when the FOSS wavelength range was restricted to match the SCiO. These results indicate that these spectrometers can acquire spectra that have good utility for predicting forage quality parameters in dried and ground samples. Finally, [10] compared the FOSS to a NeoSpectra-Scanner (1350–2550 nm, 16 nm step, Siware Systems Inc., Cairo, Egypt). They evaluated the performance of the two spectrometer systems to predict neutral detergent fiber, acid detergent fiber, acid detergent lignin, crude protein, and in vitro true digestibility using a mixed-species model over 284 alfalfa and grass samples. After trimming the instruments to the same wavelength range, the average loss in precision between the instruments was 0.14%DM.

Whereas these studies evaluated different instruments and sample sets, the loss in precision among the studies was on the same order of magnitude. However, a significant loss in precision is observed relative to standard laboratory chemical analyses. The standard error of the laboratory for forage quality parameters reported by [9] ranged from 0.21 for crude protein to 1.34 for neutral detergent fiber digestibility. Comparing the standard error of the laboratory to the standard error of prediction in that study, the predictions with the FOSS instrument were 2.6 times the laboratory compared to 3.9 for the NIRscan Nano. 

The previous literature suggests that new, low-cost near-infrared reflectance spectrometers produce forage quality prediction models with less precision. The practical implications of the loss in precision should be considered against the cost of acquiring and maintaining the devices and the sampling costs. For example, it is not practical for producers to dry and grind samples on the farm. What is the loss in precision expected when forgoing sample preparation? Other questions about the implementation of such systems remain. There is documented instrument-to-instrument variation in filter wheel and diode array benchtop NIRS systems. Still, to the authors’ knowledge, there is no publicly available information on the variation in these new devices.

Additionally, benchtop instruments control the sample presentation. However, little practical information exists on the sample presentation. In this study, we aim to answer those research questions. Specifically, we develop prediction models and report performance using a large sample database with three NeoSpectra-Scanner handheld NIRS devices on undried, unground samples.

## 2. Materials and Methods

Predictive NIRS models were developed using NIRS spectra and laboratory reference values for 555 silage samples, including alfalfa (n = 27), grass (n = 92), corn (n = 260), and mixed alfalfa and grass (n = 176) species. Silage samples were collected in 2020 and 2021 from silage bunkers on 62 farms around New York State. After collection, the samples were vacuum-packed in oxygen-limiting polyethylene bags using a commercial vacuum packing machine for scanning at a later date [3].

The acquisition of NIR spectroscopic measurement data was achieved using three NeoSpectra-Scanners (Si-Ware Systems Inc., Cairo, Egypt). The data were collected with the NeoSpectra Scan software V1.0 on an Android tablet. The device has a 10 mm collection window, and the software reports spectra from 1350 to 2550 nm at a variable step between 2.5 and 8.8 nm and a wavelength resolution of 16 nm. 

Prior to scanning, all samples were mixed well in a large plastic bin, and then samples were compressed in a rectangular box (10 × 40 cm, 13 cm deep). Two methods were used to collect spectra. For method A, the lens was placed in contact with the forage and held in place until the end of the four-second scanning period. In the second method (B), the instrument was moved across the forage sample during the four-second scanning period while maintaining contact between the instrument and the sample. The samples were remixed after each moving scan. Quadruplicate scans of the forage samples were collected using the three devices and two scan methodologies.

All calculations and analyses were performed with MATLAB version 9.8 R2020a (MathWorks, Inc., Natick, MA, USA), PLS_Toolbox version. 8.9 with MATLAB, RStudio Version 1.2.1335 (Integrated Development for R. RStudio, PBC, Boston, MA, USA), and Microsoft Excel version 16.0 (Microsoft Corporation, Redmond, WA, USA). The reflectance (R) measured by the NIR instrument was converted to absorbance values using the log (1/R) transformations. Partial least squares (PLS) regression was used to establish the relationships between responses of forage constituents and predictor variables of spectral data. The dependent variables and independent variables of the PLS calibration models were the reference values and NIR spectra, respectively. Cross-validation (CV) using the k-fold method of venetian blinds with ten data splits applied to conduct an internal validation in each calibration model. For each NIR instrument, sample scans were separated into calibration (80%) and prediction datasets (20%), and calibration samples were used to develop and cross-validate (internal) the calibration models. The remaining samples were used to predict and validate (external) the performance of the developed models. 

Pre-processing techniques, including no pre-treatment, mean center (MC), multiplicative scatter correction (MSC), Savitzky-Golay smoothing with first and second derivation (D), and combinations of any two of them were applied to process the spectral data. Calibration models were optimized based on the number of latent variables (LV) of PLS regression and different combinations of pre-processing methods. Statistical indicators of coefficient of cross-validation (R^2^CV), root mean square error of cross-validation (RMSECV), and root mean square error of prediction (RMSEP) of modeling results were considered to assess the calibration performance in this study. Significance analyses were performed using a one-way analysis of variance (ANOVA) *t*-test (*p* = 0.05).

After scanning, all samples were dried in forced-air ovens to a constant weight at 60 °C and ground in a Wiley mill (Thomas Scientific, Swedesboro, NJ, USA) to pass a 1 mm sieve and placed in labeled sealable plastic cups. Forage constituents evaluated in this study included neutral detergent fiber (NDF), in vitro digestibility (IVTD), neutral detergent fiber digestibility (NDFD), acid detergent lignin (ADL), acid detergent fiber (ADF), ash, crude protein (CP), and moisture content (MO), and all were utilized as reference data for NIRS predictive models.

Samples were analyzed using wet chemistry procedures described in [11]. Briefly, forages were weighed into ANKOM F57 filter bags (ANKOM Technology, Macedon, New York, USA) for NDF, ADF, ADL, and 48 h in vitro digestibility (IVTD) analyses. On the start and second day, filter bags were momentarily removed from the jars to express gas buildup. Neutral detergent fiber digestibility was calculated as the proportion of the total fiber digested, reported on an NDF basis.

Nitrogen (N) was determined using a combustion process (LECO CN628 analyzer, DairyOne, Ithaca, NY, USA), and crude protein (CP) was calculated as N × 6.25 (AOAC, 1995). All analyses were conducted in duplicate, except for nitrogen, which was determined in duplicate on a subset of samples to calculate a standard error of the laboratory for CP. The standard error of the laboratory (SEL) for these analyses has been reported previously [12].

## 3. Results

**Figure 1:**
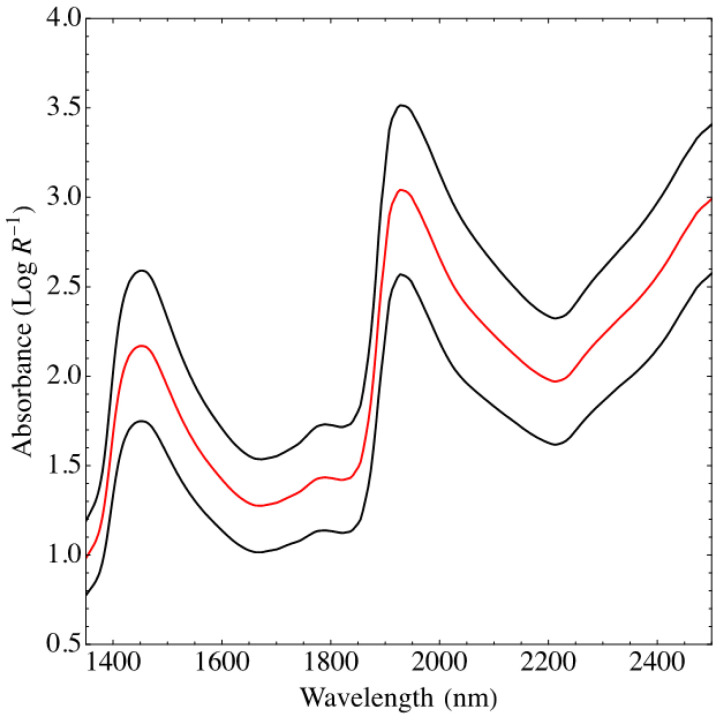
Mean (red) and standard deviation (black) of spectra collected from all forage samples using all the Neo instruments.

The mean and one standard deviation (SD) of the spectra collected from all forage samples with all three NEO spectrometers are shown in Figure 1. The two broad peaks shown at approximately 1950 nm (first overtone) and 1450 nm (O-H stretching) are assigned to O-H absorbance. A weak band around 1800 nm has been assigned to the third C-H overtone.

Chemical analyses of the 555 forage samples for neutral detergent fiber (NDF), in vitro digestibility (IVTD), neutral detergent fiber digestibility (NDFD), acid detergent lignin (ADL), acid detergent fiber (ADF), ash, crude protein (CP), and moisture content (MO) and different crop types, including alfalfa (A), grass (G), mixed alfalfa and grass (AG), and corn (C) silages are presented in Table 1. Reference values of one corn sample were missing, and further calculations associated with this corn sample were excluded. Comparing the individual species of A and G to the mixed AG, the ranges of the forage constituents were increased by combing the two species, similar to our previous study in dried and ground samples [10]. The average moisture content varied little among crop species, 60.8%, 60.2%, 62.0%, and 62.4% for A, G, AG, and C, respectively. Corn silage had the smallest variation in moisture content (SD = 6.4), which agrees with [12] and [9]. Alfalfa had the greatest average ash content (11.6%), crude protein (21.8%), and ADL (7.7%), which are significantly greater than those of corn (Ash = 3.0%, CP = 7.7%, ADL = 2.2%). Acid detergent fiber of grass was 36.9%, which is greater than that of A (34.7%), AG (34.5%), and C (20.6%). The NDF (55.5%) and NDFD (65.7%) of grass are the greatest among all crops. The NDF content of corn (34.1%) is lower than that of alfalfa (39.9%), whereas the NDFD (59.5%) of corn is greater than that of alfalfa (55.4%). The IVTD of corn was 88.4%, which is greater than that of AG (87.6%), A (83.1%), and G (82.6%). 

Near-infrared spectra were acquired using two different scanning methods in this study. Method A included taking four four-second measurements with the instrument in contact with the forage, whereas method B involved sliding the instrument across the sample during scanning. To compare the two scanning methods, models for each crop species and constituent were optimized in terms of the number of latent variables and math pretreatments as described in the materials and methods. The average RMSEP for each scanning method and constituent was computed and compiled into Table 2. The calibration models’ details for each constituent and instrument are summarized in Appendix A. Means were compared using a one-way analysis of variance, and significance was recognized at *p* < 0.05. Spectra from individual scans and instruments were treated as independent observations.

This analysis indicated that NIR spectra collected by method B resulted in better predictions of forage nutritive value. Although there was no strong evidence showing the difference between methods A and B for ADL (%DM), the *p* = 0.051 was in the margin of significance. This result indicated that data collected at position B using the NEO scanner provided more representative spectra of forage constituents. Scanning method B had a much larger contact surface that might allow the handheld instrument to obtain more accurate and precise spectra data.

Table 2 shows the validation dataset of the two calibration instruments (V1), respectively. The average calibration results of all crop constituents are shown in Table 3. The individual instrument results can be found in Appendix B. The RMSECVs of calibration models were calculated across all combinations of instruments (1&2, 2&3, or 3&1). In general, the better-performing calibration models for NDF, ADF, Ash, and CP were less susceptible to instrument variability (0.09 < *p* < 0.35). The exception was moisture content, where the average difference in RMSEP was 0.95, and the R^2^ was 0.96. ADL, IVTD, and NDFD all had poorer calibration performance R^2^ from 0.79 to 0.55 and had a tendency (IVTD) or significant (NDFD, ADL) reduction in RMSEP when the instrument in the validation set was not included in the calibration dataset.

There were four different types of crops scanned using the NEO instruments. The spectra data of all alfalfa (A), grass (G), mixed alfalfa, and grass (AG) were combined (AG_all_) and analyzed. The predictions of crop constituents using all three NEO instruments are shown in Table 4. The NEO instruments performed better when predicting forage constituents of all AG than corn by itself, according to the higher R^2^CVs (Table 4). However, the RMSEs (RMSECV, RMSEP) AG_all_ was greater than that of corn, and this may be explained by the inclusion of two different crop species of alfalfa and grass in the all AG group. 

The R^2^CVs of MO were 0.93 and 0.97 for C and AG_all_, respectively, which indicated that the NEO instrument can provide accurate predictions of moisture contents for different crops. In contrast, the NEO instrument was not accurate for predicting Ash, and the R^2^CVs of Ash were less than 0.34 for all different types of crops (Table 4). 

Regardless of crop species, models for NDFD and Ash explained less than half of the variation in these constituents. This is also true for IVTD, ADL, and CP in the corn samples. NDF, ADF, and CP were better predicted in the AG_all_ samples with R^2^CVs ranging from 0.81, 0.74, and 0.67, respectively. However, if one were to convert these data to the ratio of performance to deviation (RPD = 1/√(1 − R^2^)), all of these constituent prediction models would fall under the “Very poor—not recommended” categorization established by [13]. The moisture prediction model would meet the criterion of “Fair—Screening” for corn and “Excellent—Any application to this type of material” for the AG_all_ model.

Rukundo et al. [14] observed similar results for the forage quality parameters of ADF and ADL in switchgrass samples. Still, their result was more promising for in vitro dry matter digestibility (IVDMD), which is analogous to IVTD. Still, their samples varied more, having a standard deviation of 9.93 compared to 3.8 and 3.1 for the AG_all_ and corn datasets of this study, respectively. Acosta et al., Berzaghi et al., and Digman et al. [8,9,10] all reported better results than those presented here, but only one spectrometer was used in these cases, and the samples were dried and ground. Cherney et al. [12] evaluated a handheld diode array spectrometer in undried and unground forages. They reported it to have poor performance in predicting NDF, ADF, CP, Ash, and DM for individual ensiled crop species and total mixed rations using vendor-supplied calibrations. However, the exception was DM, where the RPD was 1.7.

## 4. Conclusions

Three handheld NIR spectrometers predicted moisture contents at a quantitative level on undried and unground alfalfa and grass silage samples. The prediction performance was susceptible to how the sample was presented with sliding scans outperforming stationary. The models also performed better when the same instrument was used in the calibration and validation data sets. Corn silage moisture predictions had a lower level of performance and were classified as useful for screening. All other combinations of crops and constituents had lower performance. Future work should focus on evaluating methods to mitigate instrument-to-instrument differences and to understand the utility of a screening level of performance NIRS in predicting forage quality.

## Figures and Tables

**Table 1:** Descriptive statistics for ensiled forage samples used for model development and validation in this study.

Constituent	Crop	N	Mean	SD	Range
MO (%w.b.)	A	27	60.8	8.7	39.5–75.5
	G	92	60.2	11.6	28.5–77.6
	AG	176	62.0	9.2	26.3–81.7
	C	259	62.4	6.4	29.9–82.2
Ash (%DM)	A	27	11.6	1.7	8.5–14.9
	G	92	9.6	2.3	5.3–19.9
	AG	176	11.0	2.1	3.1–21.7
	C	259	3.0	0.56	0.7–4.5
CP (%DM)	A	27	21.8	2.2	18.2–27.1
	G	92	15.3	3.5	8.8–23.0
	AG	176	19.9	3.2	10.2–27.5
	C	259	7.7	0.79	4.0–10.3
NDF (%DM)	A	27	39.9	4.0	32.6–49.1
	G	92	55.5	8.0	41.9–72.0
	AG	176	45.5	7.3	28.8–70.7
	C	259	34.1	4.6	25.4–57.4
NDFD (%DM)	A	27	55.4	7.7	44.6–72.5
	G	92	65.7	9.3	44.1–83.1
	AG	176	64.7	8.3	46.3–83.3
	C	259	59.9	4.7	44.2–78.2
IVTD (%DM)	A	27	83.1	4.1	73.0–89.5
	G	92	82.6	7.1	68.9–92.9
	AG	176	87.6	3.8	66.3–94.8
	C	259	88.4	3.1	77.8–94.9
ADL (%DM)	A	27	7.7	1.6	4.7–11.3
	G	92	6.4	2.1	2.6–15.9
	AG	176	6.6	1.6	3.2–12.2
	C	259	2.2	0.45	1.2–3.5
ADF (%DM)	A	27	34.7	3.6	29.8–43.5
	G	92	36.9	5.5	27.5–48.4
	AG	176	34.5	5.1	21.5–50.1
	C	259	20.6	2.9	15.3–33.9

N—number of samples, SD—standard deviation of the sample set, %w.b.—percent wet basis, % DM—percent dry matter, MO—moisture content, NDF—neutral detergent fiber, IVTD—in vitro true digestibility, NDFD—neutral detergent fiber digestibility, ADF—acid detergent fiber, ADL—acid detergent lignin, CP—crude protein.

**Table 2:** The PLS calibration results of RMSEPs of two scanning methods—A four-second stationary, B four-second sliding.

Scanning Method	RMSEP		*p* Value
	A	B	
NDF	4.7	4.0	0.004
IVTD	3.4	2.9	0.032
NDFD	6.1	5.2	0.033
ADL	1.2	1.0	0.051
ADF	3.8	3.1	0.025
Ash	1.5	1.3	0.012
CP	1.9	1.6	0.017
MO	1.9	1.4	0.002

RMSEP—root mean standard error of prediction, NDF—neutral detergent fiber (%DM), IVTD—in vitro true digestibility (%DM), NDFD—neutral detergent fiber digestibility (%DM), ADL—acid detergent lignin (%DM), ADF—acid detergent fiber (%DM), CP—crude protein (%DM), MO—moisture content (%w.b.).

**Table 3:** The average number of latent variables, root mean standard error of calibration, and prediction when two instruments were used for both calibration and validation (V1) and when the two instruments used for calibration were excluded from the validation set (V2).

Constituent	RMSECV	R^2^CV	RMSEP		*p* Value
			V1	V2	
NDF	4.06	0.84	4.05	4.08	0.351
IVTD	3.26	0.55	2.99	5.30	0.065
NDFD	6.40	0.29	5.73	7.21	0.024
ADL	1.17	0.79	1.03	1.18	0.035
ADF	3.15	0.84	3.13	3.31	0.085
Ash	1.78	0.82	1.52	3.06	0.096
CP	1.78	0.92	1.78	2.18	0.103
MO	1.75	0.96	1.44	2.39	0.018

RMSECV—root mean standard error of cross-validation, R^2^CV—average of the coefficient of determination for cross-validation sets, RMSEP—root mean standard error of prediction, NDF—neutral detergent fiber (%DM), IVTD—in vitro true digestibility (%DM), NDFD—neutral detergent fiber digestibility (%DM), ADL—acid detergent lignin (%DM), ADF—acid detergent fiber (%DM), CP—crude protein (%DM), MO—moisture content (%w.b.).

**Table 4:** Comparison of nutritive constituent predictions of NEO scanner between corn and mixed alfalfa and grass species.

Constituents	Crop	RMSECV	RMSEP	R^2^CV
NDF	C	3.17	3.22	0.52
	AG_all_	3.97	4.18	0.81
IVTD	C	2.25	2.21	0.44
	AG_all_	3.67	3.86	0.58
NDFD	C	4.48	4.55	0.08
	AG_all_	6.04	6.14	0.56
ADL	C	0.40	0.38	0.21
	AG_all_	1.29	1.10	0.52
ADF	C	2.11	2.07	0.45
	AG_all_	2.69	2.75	0.74
Ash	C	0.51	0.50	0.20
	AG_all_	1.93	1.42	0.33
CP	C	0.58	0.51	0.48
	AG_all_	2.27	2.71	0.67
MO	C	1.68	1.62	0.93
	AG_all_	1.78	1.67	0.97

RMSECV—root mean standard error of cross-validation, R^2^CV—average of the coefficient of determination for cross-validation sets, RMSEP—root mean standard error of prediction, NDF—neutral detergent fiber (%DM), IVTD—in vitro true digestibility (%DM), NDFD—neutral detergent fiber digestibility (%DM), ADF—acid detergent fiber (%DM), ADL—acid detergent lignin (%DM), CP—crude protein (%DM), MO—moisture content (%w.b.).

## Data Availability

The data presented in this study are available on request from the corresponding author.

## Appendix A

**Table A1:** The number of latent variables (LV), root mean standard error of cross-validation (RMSECV), and prediction (RMSEP) results of three NeoSpectra-Scanner handheld instruments (NEO) using two scanning positions for predicting forage constituents.

Constituents	Inst.	Preprocessing	LVs	RMSECV	RMSEP	R^2^CV
NDF	A-1	MC, D-2,2,15	7	4.90	4.87	0.76
	A-2	MC, D-2,2,15	8	4.78	4.49	0.77
	A-3	D-2,2,15	7	5.52	4.83	0.70
	B-1	MC, D-2,2,15	8	3.70	3.89	0.86
	B-2	MC, D-2,2,15	7	4.19	4.06	0.83
	B-3	MSC, D-2,2,15	8	4.13	3.93	0.83
IVTD	A-1	MC, D-2,2,15	6	3.39	3.31	0.51
	A-2	MC, D-1,2,15	7	3.41	3.38	0.50
	A-3	MC, D-2,2,15	6	3.50	3.45	0.48
	B-1	MC, D-1,1,15	7	3.32	3.18	0.53
	B-2	MSC, D-2,2,15	7	3.18	2.72	0.57
	B-3	MC, D-1,1,15	7	3.31	2.77	0.53
NDFD	A-1	MSC, D-2,2,15	6	6.18	5.79	0.32
	A-2	MC, D-1,2,15	7	6.41	6.00	0.27
	A-3	MC, D-2,2,15	6	6.23	6.37	0.32
	B-1	MC, D-2,2,15	8	5.43	4.79	0.48
	B-2	MC, D-2,2,15	8	5.76	5.38	0.42
	B-3	MC, D-2,2,15	7	5.49	5.43	0.47
ADL	A-1	MSC, D-2,2,15	7	1.32	1.25	0.74
	A-2	MSC, D-2,2,15	8	1.29	1.15	0.75
	A-3	MSC, D-2,2,15	7	1.36	1.10	0.72
	B-1	D-1,2,15	7	1.15	0.97	0.80
	B-2	MSC, D-2,2,15	7	1.19	1.08	0.79
	B-3	MC, D-2,2,15	6	1.19	0.97	0.79
ADF	A-1	MC, D-2,2,15	6	3.91	4.08	0.79
	A-2	MSC, D-2,2,15	8	3.55	3.47	0.82
	A-3	D-2,2,15	7	4.23	3.80	0.75
	B-1	MC, D-2,2,15	7	3.00	2.89	0.87
	B-2	MC, D-2,2,15	7	3.39	3.25	0.84
	B-3	MC, D-2,2,15	8	3.18	3.03	0.86
Ash	A-1	MC, D-1,2,15	6	1.82	1.49	0.81
	A-2	D-1,2,15	6	1.91	1.53	0.79
	A-3	MC, D-1,1,15	9	1.78	1.47	0.82
	B-1	MC, D-1,1,15	7	1.52	1.30	0.87
	B-2	MC, D-1,1,15	8	1.60	1.33	0.85
	B-3	MC, D-1,2,15	8	1.55	1.17	0.86
CP	A-1	D-1,1,15	6	2.03	1.99	0.89
	A-2	D-1,1,15	8	1.93	1.77	0.90
	A-3	MC, D-1,1,15	9	2.03	1.86	0.89
	B-1	MC, D-1,2,15	7	1.57	1.62	0.94
	B-2	MC, D-1,2,15	8	1.68	1.55	0.93
	B-3	D-1,1,15	6	1.66	1.63	0.93
MO	A-1	MSC, D-1,2,15	6	2.15	2.00	0.94
	A-2	MSC, D-1,2,15	6	2.16	1.76	0.94
	A-3	MSC, D-2,2,15	6	2.08	1.87	0.94
	B-1	MC, D-1,1,15	5	1.73	1.40	0.96
	B-2	MSC, D-1,1,15	7	1.68	1.32	0.96
	B-3	D-1,1,15	5	1.81	1.33	0.96

MC—mean center, D—Savitzky-Golay smoothing with first and second derivation (derivative order—1st or 2nd, polynomial order—first (1) or second (2), and number of wavelengths), MSC—multiplicative scatter correction; A—stationary scanning, B—sliding scanning.

## Appendix B

**Table A2:** The number of latent variables (LV), root mean standard error of cross-validation (RMSECV), and prediction (RMSEP) when two instruments (designated 1, 2, or 3) were used for both calibration and validation (V1) and when the two instruments used for calibration were excluded from the validation set (V2).

	Calibration	V1	V2	LVs	RMSECV	RMSEP	R^2^CV	Avg RMSEP	*p*-Value
NDF	1&3		2	6	3.99	4.14	0.84	4.08	0.351
	1&2		3	5	4.15	4.09	0.83		
	2&3		1	5	4.14	4.02	0.83		
		1&3		6	3.86	3.88	0.85	4.05	
		1&2		5	4.12	4.12	0.83		
		2&3		5	4.14	4.14	0.83		
IVTD	1&3		2	9	3.31	4.69	0.53	5.30	0.065
	1&2		3	8	3.26	3.58	0.55		
	2&3		1	9	3.22	7.63	0.56		
		1&3		9	3.31	3.09	0.53	2.99	
		1&2		8	3.26	3.05	0.55		
		2&3		9	3.22	2.84	0.56		
NDFD	1&3		2	5	6.36	6.72	0.28	7.21	0.024
	1&2		3	8	6.79	6.76	0.25		
	2&3		1	6	7.16	8.15	0.14		
		1&3		5	6.25	6.08	0.31	5.73	
		1&2		8	5.67	5.26	0.43		
		2&3		6	6.16	5.83	0.33		
ADL	1&3		2	8	1.10	1.14	0.82	1.18	0.035
	1&2		3	6	1.19	1.24	0.79		
	2&3		1	6	1.22	1.17	0.78		
		1&3		8	1.09	0.93	0.82	1.03	
		1&2		6	1.19	1.08	0.79		
		2&3		6	1.23	1.09	0.78		
ADF	1&3		2	6	3.07	3.46	0.87	3.31	0.085
	1&2		3	6	3.21	3.30	0.86		
	2&3		1	6	3.17	3.16	0.86		
		1&3		6	3.07	3.01	0.87	3.13	
		1&2		6	3.21	3.23	0.86		
		2&3		6	3.17	3.14	0.86		
Ash	1&3		2	5	1.70	2.07	0.83	3.06	0.096
	1&2		3	4	1.79	2.08	0.82		
	2&3		1	6	1.97	5.03	0.78		
		1&3		5	1.70	1.50	0.83	1.52	
		1&2		4	1.79	1.53	0.82		
		2&3		6	1.71	1.52	0.83		
CP	1&3		2	8	1.84	2.68	0.91	2.18	0.103
	1&2		3	8	1.69	1.90	0.93		
	2&3		1	7	1.98	1.95	0.90		
		1&3		8	1.68	1.83	0.93	1.78	
		1&2		8	1.69	1.65	0.93		
		2&3		7	1.80	1.87	0.92		
MO	1&3		2	7	1.60	3.00	0.97	2.39	0.018
	1&2		3	8	2.02	2.06	0.95		
	2&3		1	7	2.00	2.10	0.95		
		1&3		7	1.60	1.43	0.97	1.44	
		1&2		8	1.62	1.42	0.96		
		2&3		7	1.64	1.47	0.96		

## References

[1] St-Pierre N.R., Weiss W.P. (2015). Partitioning Variation in Nutrient Composition Data of Common Feeds and Mixed Diets on Commercial Dairy Farms. J. Dairy Sci..

[2] Turiello P., Larriestra A., Bargo F., Relling A., Weiss W. (2018). Sources of Variation in Corn Silage and Total Mixed Rations of Commercial Dairy Farms. Prof. Anim. Sci..

[3] Cherney D.J.R., Digman M., Cherney J.H. (2021). Day-to-Day Variation in Forage and Mixed Diets in Commercial Dairy Farms in New York. Appl. Anim. Sci..

[4] Yoder P.S., St-Pierre N.R., Daniels K.M., O’Diam K.M., Weiss W.P. (2013). Effects of Short-Term Variation in Forage Quality and Forage to Concentrate Ratio on Lactating Dairy Cows. J. Dairy Sci..

[5] Williams P., Norris K. (2001). Near-Infrared Technology: In the Agricultural and Food Industries.

[6] Beć K.B., Grabska J., Siesler H.W., Huck C.W. (2020). Handheld Near-Infrared Spectrometers: Where Are We Heading?. NIR News.

[7] Fernández Pierna J.A., Vermeulen P., Lecler B., Baeten V., Dardenne P. (2010). Calibration Transfer from Dispersive Instruments to Handheld Spectrometers. Appl. Spectrosc..

[8] Acosta J.J., Castillo M.S., Hodge G.R. (2020). Comparison of Benchtop and Handheld Near-infrared Spectroscopy Devices to Determine Forage Nutritive Value. Crop Sci..

[9] Berzaghi P., Cherney J.H., Casler M.D. (2021). Prediction Performance of Portable near Infrared Reflectance Instruments Using Preprocessed Dried, Ground Forage Samples. Comput. Electron. Agric..

[10] Digman M.F., Cherney J.H., Cherney D.J.R. (2022). The Relative Performance of a Benchtop Scanning Monochromator and Handheld Fourier Transform Near-Infrared Reflectance Spectrometer in Predicting Forage Nutritive Value. Sensors.

[11] Valentine M.E., Karayilanli E., Cherney J.H., Cherney D.J. (2019). Comparison of in Vitro Long Digestion Methods and Digestion Rates for Diverse Forages. Crop Sci..

[12] Cherney J.H., Digman M.F., Cherney D.J. (2021). Handheld NIRS for Forage Evaluation. Comput. Electron. Agric..

[13] Williams P. (2014). The RPD Statistic: A Tutorial Note. NIR News.

[14] Rukundo I.R., Danao M.-G.C., Mitchell R.B., Masterson S.D., Weller C.L. (2021). Comparing the Use of Handheld and Benchtop NIR Spectrometers in Predicting Nutritional Value of Forage. Appl. Eng. Agric..

